# Knowledge, Perception, and Practices Concerning African Swine Fever in Smallholder Pig Value Chain in North Central Nigeria: Implications for Adaptation of Prevention and Control

**DOI:** 10.1155/tbed/5582374

**Published:** 2025-08-28

**Authors:** Victoria Isioma Ifende, Rebecca Weka, Vincent B. Muwanika, Matthew Y. Gukut, Pam D. Luka, Charles Masembe, Erika Chenais

**Affiliations:** ^1^College of Natural Sciences, Makerere University, Kampala, Uganda; ^2^Veterinary Extension Services Department, National Veterinary Research Institute, Vom, Nigeria; ^3^College of Agriculture and Environmental Sciences, Makerere University, Kampala, Uganda; ^4^Biotechnology Centre, National Veterinary Research Institute, Vom, Nigeria; ^5^Department of Epidemiology, Disease Surveillance and Risk Assessment, Swedish Veterinary Agency, Uppsala, Sweden; ^6^Department for Animal Biosciences, Swedish University of Agricultural Sciences, Uppsala, Sweden

**Keywords:** African swine fever, Jos, Nigeria, participatory approach, perception, pig value chain, smallholder farmers

## Abstract

African swine fever (ASF) is a devastating disease of pigs that is endemic in Nigeria. Smallholder farmers have been implicated in driving disease spread, yet little is known about their perceptions about the disease and the role they play in the local epidemiology. Additionally, it remains inadequately documented how other smallholder pig value chain actors perceive and influence ASF spread. This study investigated the perceptions and practices of smallholder pig value chain actors concerning ASF by conducting 62 focus group discussions (FGDs) consisting of 516 participants. Participants included pig farmers and other value chain actors in separate interviews, purposively selected from pig-producing communities with previous laboratory confirmation of ASF. Participatory epidemiology (PE) tools were used to investigate perceptions of clinical signs, transmission routes, occurrence, and control, as well as practices relating to ASF. The most frequently reported clinical signs of ASF were inappetence and red skin/spots. Most frequently mentioned routes of spread were air and farm visits. Most of the other value chain actors, apart from farmers, believed that they had a responsibility for controlling ASF. Seasonal calendars from both participant categories indicated that peak occurrence of outbreaks coincided with the rainy season, when the animals are confined. Practices reported by both categories included sale of sick pigs and improper disposal of dead pigs and slaughter remains, which could facilitate ASF spread. Both participant categories showed limited knowledge of disease control. Our findings provide insights about the local epidemiology of ASF in the smallholder pig value chain in Nigeria, indicating the role of indirect transmission of ASF. The reported temporal patterns and the potential role of butchers and traders in ASF spread further show the importance of investigating the local disease context in different settings to be able to provide relevant advice for mitigation strategies.

## 1. Introduction

Pig production contributes to rural and peri-urban livelihoods in many parts of Africa [1–3]. In West Africa, Nigeria has the highest population of pigs, estimated to be over seven million [4], with more pigs in the North than in the South of the country [5]. Pigs play an important role in the social and cultural life of many pig-keeping communities in these areas [6, 7]. They are sold for purchase of household goods, to celebrate festivities, and to fulfill dowry obligations. The value chain is mostly informal, with pigs sold directly to butchers or traders who may sometimes also serve as middlemen.

African swine fever (ASF), a viral, deadly pig disease, poses a challenge to the development of the pig industry in Nigeria, with an estimated annual disease burden of 6.2 million USD (1.2 billion Naira) attributed to pig deaths [8]. ASF is further estimated to be a threat to the food security of over 200 million Nigerians [9]. In Nigeria, transmission has been attributed to the domestic pig cycle, involving direct and indirect transmission between pigs and pig products [7, 10]. In this cycle, human activities further drive virus transmission [11, 12]. There is still no commercially available vaccine for ASF of the genotypes present in Nigeria [13–15].

The first outbreaks of ASF in Nigeria were reported in the southern parts of the country in 1997, with spread towards the North reported from 1998 [16, 17]. After two decades of ASF presence, the disease is now endemic. Most of the outbreaks occurring are not formally reported or subject to epidemiological investigation [17, 18]. A few studies have identified risk factors for virus transmission, such as presence of an abattoir in the farming community, presence of infected farm in the neighborhood, visits by vets/paravets, external source of replacement stock, and dry season [10, 19]. Most of these studies have been carried out with farmers in the commercial pig value chain in the southern part of Nigeria, yet the pig sector in Nigeria is dominated by smallholder farmers. In north central Nigeria, Plateau State is a major hub for smallholder pig farming, with two live pig markets [20], and endemic occurrence of ASF since 2001 [10, 17, 21]. However, there is limited information about participants in the value chain and their role in the spread of ASF. There are thus still many gaps in our understanding of the epidemiology of ASF in Nigeria. To develop effective disease control strategies, it is pertinent to improve the understanding of the value chain and actors, including their perceptions of the disease and the control, as well as the motivations for current practices. Participatory epidemiology (PE) has been advocated as a suitable method in this regard [22]. The objective of this study was to investigate the knowledge, perceptions, and practices of smallholder pig value chain actors related to ASF in North-Central Nigeria, with the purpose of improving the control of the disease.

## 2. Materials and Methods

### 2.1. Study Area

**Figure 1:**
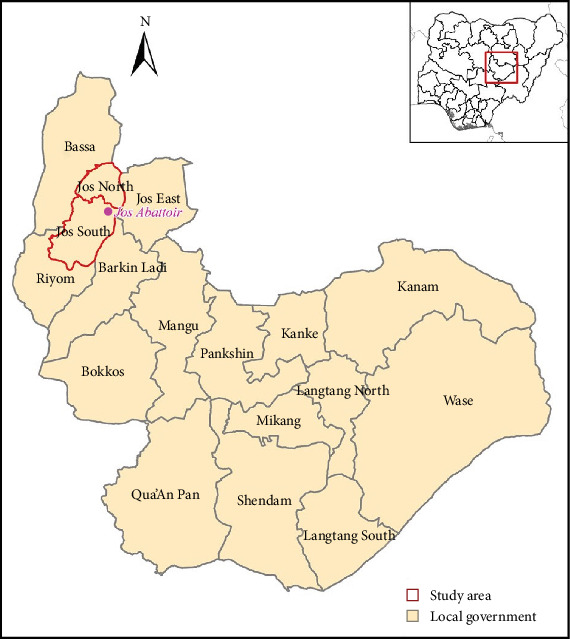
Map of Plateau State showing the study area in and around Jos, North Central Nigeria.

The study was carried out in five districts (Gwong, Du, Gyel, Kuru, and Vwang) in and around Jos, the administrative capital of Plateau State in North central Nigeria (Figure 1). Plateau State has a near temperate climate with mean temperature of between 18°C and 22°C, and harmattan winds causing cold weather between December and February. The peak of rainfall is recorded during the months of July and August [23]. The state is home to over 4.2 million people belonging to 40 ethno-linguistic groups [24]. The indigenous language in Jos is Berom; however, common languages are Hausa and English. The major economic activity in Plateau State is agriculture, it is further known as a major pig producing area with an estimated pig population of 1,725,562 pigs [5].

### 2.2. Study Design and Participant Selection

A qualitative interview study with methods from PE was applied in 62 focus group discussions (FGDs) carried out between September and November 2019. Two participant categories of FGDs were organized, one for pig farmers (hereafter called farmer FGD) and one for other value chain actors that included butchers, middlemen (traders of live pigs), and pork sellers (hereafter called trader FGD). The five districts were purposively selected based on the large number of pig farmers, a long tradition of smallholder farmers keeping pigs, and previously reported occurrence of ASF [17, 18, 25].

In each of the five districts, three interview sites were selected, except in Kuru, where only one site was included due to the small size of the district. The study sites were purposively selected based on large number of smallholder pig farmers and other value chain actors. The site selection process included consultations with veterinary authorities and key informants such as leaders of pig farmers' and butchers' associations. In each selected site, between two and six FGDs were held depending on relative density of pig farmers in the villages around the site. Participants for the farmer FGDs were purposively selected based on lists generated via district heads or contact persons within and around the interview sites. Only farmers having not more than 50 pigs were invited; attention was paid to include both male and female participants. Selected participants were invited by the key informants.

Actors from the pig production value chain other than farmers included people that had their businesses located at Jos abattoir, as well as at pork selling and slaughter points in and around the selected sites (see Table 1). For the abattoir, the groups were convened at their convenience by recruiting value chain actors that were available at the time of visit following prior agreed appointment. The other value chain actors were recruited purposively based on their activities in the value chain and association with selected sites. These other value chain actors will hereafter be referred to as “traders”.

### 2.3. Data Collection

The research team consisted of total two facilitators and two note takers divided in two teams. All were trained in participatory methods and research ethics before the study. Each team included at least one person proficient in Hausa and Berom languages, respectively and all were proficient in English. The FGDs followed a predefined checklist adapted from a study by Chenais et al. [26], modified to suit the local circumstances and specific objectives of this study. Two pretests with subsequent adjustment of the checklist were done prior to the study. At the start of each FGD, the participants were informed about the study objectives, particularly stating that it was research, without immediate benefits for the participant. Participants were informed that they could join or leave at any time or choose not to answer specific questions. Oral consent to participate in the study and for audio recordings and photographs was assured from all participants; after which a background data sheet was used to collect demographic details (age, gender, educational status, herd size). The participants were asked to select a common language (English or Hausa) for the FGD. Notes were taken in English. In a very few cases where an individual within the group understood but did not speak English or Hausa, this person spoke Berom while the main discussion was in English or Hausa. The facilitator confirmed responses in Hausa, and note taker still took the notes in English. The PE tools used included listing, hand-counts, proportional piling, and seasonal calendars [26, 27], see Table 2. Beans were used as counters for the seasonal calendar. The facilitator paid specific attention to include all participants in the discussion. The checklist consisted of open-ended questions allowing participants to steer the discussion and covered areas, such as knowledge of ASF (name used in local languages, clinical signs, age and breed affected, occurrence); perceptions of route of spread and control of the disease; practices employed during ASF outbreaks as well as data on the daily pig husbandry situation (sources of stocking/restocking, sale and slaughter points). Each farmer FGD commenced with a warm-up question inquiring about the participants' purpose for keeping pigs and management system; trader FGDs started with inquiring which locations they sourced their pigs from. Management system herein refers to the farmer's provision of feed, health care, and housing to pigs. Extensive system was defined as nonprovision of the above (i.e., the traditional free-roaming/free-range and scavenging pigs), semi-intensive system was defined as partial confinement and provision of feed and healthcare, while intensive system here referred to permanent confinement of pigs and provision of feed and healthcare. Right after the warm-up questions, participants in each category were asked to list common pig diseases that have affected their pigs, with observed signs; after which the facilitator identified the disease (together with the participants and first author) that fitted the commonly accepted syndromic case description of ASF (e.g., anorexia, reduced general condition, fever/huddling together, unwillingness to move, diarrhea/vomiting, red skin, sudden death, high mortality, and morbidity plus signs of the disease being infectious) [28] and narrowed down further questioning to this disease. An indication of red skin, sudden death and high mortality were particularly considered suggestive of ASF amongst/other signs listed above. From this point on in the discussion, the facilitator referred to the disease being discussed using the most commonly used local name by the respective group/as ASF. Participants were thereafter asked to create a seasonal calendar using proportional piling across the 12 months of a year. The seasonal calendar covered the following topics: rainfall, occurrence of ASF, and pig/pork sales for both farmer FGDs and trader FGDs, and in addition, “confinement of pigs” and “presence of wild pigs” for farmers. Participants were asked to consider the past 2 years while constructing the calendar. This time span was chosen to avoid recall bias. No external event was used to define the time period. Consensus from all group participants on the allocation of beans was sought before the final results were recorded. In FGDs where participants did not respond to a question even after probing, this was noted as “could not answer”. Discussions were captured by taking notes, in addition to key points that were noted on flip charts following consensus agreement by the group. To understand the husbandry system and to triangulate data, direct observations in the form of farm visits were done whenever possible.

### 2.4. Data Analysis

Quantitative and semi-quantitative data were entered into Microsoft Excel spreadsheet (Microsoft Redmond, WA, USA). Responses were collated with the FGD as unit of observation in most cases, and reported as proportions of FGDs giving each response. For questions answered with listings, this meant that if, for example, one individual participant mentioned a clinical sign of ASF, it was recorded that the group was aware of that clinical sign. In the same way, hand counts were used to identify presence of practices on group level, not to calculate individuals using a specific practice. Hand count of at least 20% of the group was reported as presence of practice. If less, it was reported as “mentioned”.

However, demographic and some background data were reported with the individual participant as the unit of observation using descriptive statistics calculated with IBM Statistical Products and Services Solution (SPSS version 25, Armonk, New York, USA). The data from the seasonal calendar were summarized as mean values for each variable and plotted as graphs using Microsoft Excel.

## 3. Results

A total of 62 FGDs with 516 participants (on average eight, minimum [min] six, maximum [max] 10 per FGD) from 30 different villages were included in the study. The participants included 452 farmers and 64 traders, with the latter group representing middlemen, butchers, pork kiosk owners, pork joint owners (traders of cooked/roasted pork), and slaughterers. The FGDs lasted on average 75 min (min–max 50–90 min).

### 3.1. Demographic and Background Information

A total of 239 (46%) of the participants were female. The average age was 43 years (min–max 18–100 years). 74 (14%) of the participants had no formal education, and 421 (81%) had at least primary school education. Participant farmers kept an average of five pigs (min–max 1–50). Among the traders, most of the butchers slaughtered fewer than seven pigs per day (min–max 1–7). Most farmers (*n* = 452, 95%) reported that their pigs were housed, 332 farmers (74%) practiced an intensive (confined) system of management, no farmer reported practicing an extensive management system (absolute free roaming of scavenging pigs without provision of food and health care), see Table 3. However, some farmers added that they sometimes allow their pigs to roam in the dry season because of inadequate money to provide enough feed for the pigs, while some tethered the pigs during the rainy season when housing pens got demolished and funds for repairs were scarce. All pigs were confined during the rainy season in order to protect agricultural crops. This custom was further passed as community law in most communities. Most farmers (*n* = 452, 80%) both bred and fattened pigs. 327 (72%) of the farmers kept local breeds.

Most of the traders performed several activities, with over half (*n* = 64, 57%) of them working as butchers. Butchers usually bought live pigs and slaughtered to sell, while some butchers were also involved in live pig trading. The slaughterers rendered service in slaughtering of pigs bought by individuals for consumption or for pork sale. Twenty (31%) of the traders owned and bred pigs, see Table 3.

Most farmer FGDs reported feeding pigs with self-compounded feed and swill. A few farmers indicated feeding their pigs with commercial feed, while 24 of the 54 FGDs (44%) had some farmers who mixed self-compounded feed with commercial feeds. Poultry litter was also reportedly used as feed for pigs.

Reasons given for keeping pigs included income generation used for cash payments such as school fees, medical bills, food items, building of houses, dowry, land, rent, fertilizer, and farm inputs. Pigs were also kept for pork consumption, manure production, trade, and savings as well as love for farming. Pig farming was considered less risky than poultry farming. Additional benefits reported by farmers included pig fat used for frying, and feeding pigs with food leftovers as means of converting waste.

The traders listed more than 20 areas in and around Jos, other towns in Plateau State as well as Bauchi in a neighboring state from where they sourced pigs. Sources included farms and live pig markets. In one of the FGDs, the participants indicated that they were involved in all activities in the pig value chain.

### 3.2. Knowledge

#### 3.2.1. Local Names

A total of 15 local names were listed for ASF, with “Robi alede” (meaning “disease of pigs” in Berom) having highest frequency with listings in five FGDs; others were “Ciwon alede” (“disease of pigs” in Hausa) and skin disease (Table 4). The names were mostly descriptions of clinical signs and were not the same for the different FGDs within the same district, and sometimes not the same amongst the participants in the same FGD. The names adopted by the participants did not necessarily reflect the languages they spoke. Some of the farmer FGDs described common signs of ASF and called it a “dreaded disease of pigs”. In 18 farmer FGDs, the farmers did not have a specific name for ASF. In the trader FGDs, the listed names within the FGDs were always common to all participants within the groups. The butchers at the abattoir in Jos knew ASF as “Siriri”, a word they reported was made up by them to describe “shivering” in sick pigs.

#### 3.2.2. Clinical Signs

The farmer FGDs listed a total of 41 different clinical signs of ASF, with every FGD reporting at least two clinical signs. The most frequently mentioned clinical sign was “animal being off feed” (39 FGDs); red skin, death, weakness, and shivering were listed in 38, 31, 24, and 21 FGDs, respectively (Table 4 and Table S1 for complete list). The presence of flies was listed as a clinical sign for ASF in six groups. Farmers in 24 FGDs (44%) indicated they were certain to recognize an ASF-infected pig if they saw the signs, while 22 FGDs could not answer this question. In most FGDs (63%), farmers indicated that both piglets and adults were affected by the disease. A total of 16 clinical signs were listed by trader FGDs, including inappetence and red spots on the skin, which were mentioned in all trader FGDs (Table 4). These participants also listed a total of 10 signs they could see in pork from ASF-infected pigs, the most frequently mentioned sign being hemorrhage in internal organs (Table S1). All participants in the trader FGDs reported that they could recognize signs of ASF and had seen ASF in pigs and in pork.

#### 3.2.3. Routes of ASF Spread

A total of 43 routes of spread were mentioned in the farmer FGDs (Table S1). Most frequently mentioned were airborne (20 FGDs, 37%), visiting infected farms (10 FGDs, 19%), and pig-to-pig contact (10 FGDs). Most FGDs mentioned between two and four routes. In seven FGDs, this question was not answered, while in three FGDs, the farmers said they did not know what routes introduced the disease. There was no mention of wild pigs transmitting ASF.

A total of 11 routes of spread for ASF were mentioned by the participants of trader FGDs. Most trader FGDs mentioned “air” to be the route of spread (See Table 4). More than half of the trader FGDs (5 FGDs, 63%) mentioned “farm to farm” as a route. Most of the trader FGDs (7 FGDs, 88%) mentioned three or more routes of infection.

### 3.3. Perception

In most (30 FGDs, 55%) farmer FGDs, there were participants who either stated that they were unaware (13 FGDs, 24%) or did not respond to the question (17 FGDs, 31%), on whether ASF poses a public health risk; while most (five FGDs, 62%) of the trader FGDs stated that ASF does not pose a public health risk, see Table 5. When asked if they knew a model farmer, 14 FGDs (26%) had farmers who knew a model farmer. Participants listed confinement of pigs, good feeding of pigs, and good hygiene as reasons for success of the model farmers. During these discussions, some farmer FGDs blamed “butchers” for spreading ASF.

In more than half (five FGDs, 62%) of the trader FGDs, at least one participant was aware that their business activities could spread ASF. Six trader FGDs (75%) included actors who thought they had a responsibility in the control of ASF in relation to their activities. Business activities mentioned in this regard were selling sick pigs, consuming infected pigs, improper disposal of blood and remains of carcass after slaughter, and careless handling of blood and offal. In these six FGDs, all participants agreed that they had a responsibility to control the spread. In addition, some of the participants in these six FGDs mentioned that they avoided farm visits and mating of the pigs during outbreaks. The participants in the remaining FGDs which did not agree it was their own responsibility, stated that it was the responsibility of the veterinarians to control ASF.

### 3.4. Seasonal Occurrence of ASF

**Figure 2:**
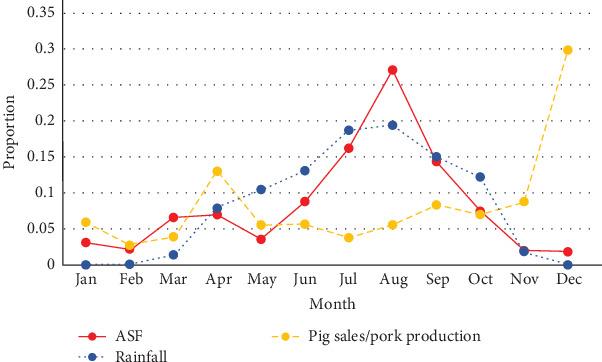
Seasonal occurrence of African swine fever, rainfall, and pig sales/pork production as reported by farmer focus group discussions in and around Jos, North Central Nigeria.

**Figure 3:**
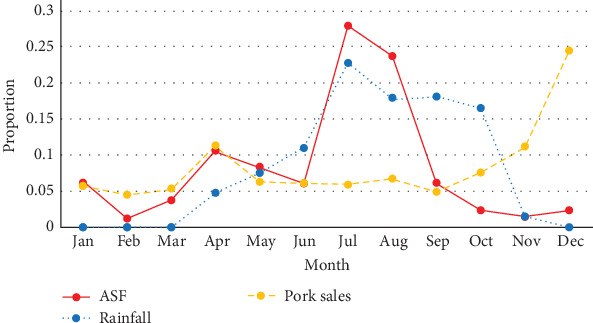
Seasonal occurrence of African swine fever, rainfall, and pork sales as reported by traders focus group discussions in and around Jos, North Central Nigeria.

ASF outbreaks were reported to most frequently occur in July and August. Some farmer FGDs associated ASF outbreaks with the rainy season, see Figure 2. The trader FGDs reported an increase in ASF outbreaks in April (Figure 3), with peak occurrence in July. The seasonal occurrence of pig sales as reported by the farmer FGDs did not show any co-occurrence with the seasonal peak occurrence of the outbreaks. The peak of pig sales was reported to be towards the end of the year (November–December), attributed to Christmas season and mining periods in some locations. “High sales” was also mentioned in April due to Easter and for buying farm inputs in preparation for the farming season. Results from the trader FGDs and farmer FGDs showed similar patterns for both ASF occurrence as well as pig and pork sales. The trader FGDs also reported that the rise in sales in October was due to harvest season, which comes with some postharvest celebrations. All 62 FGDs affirmed the absence of wild pigs. Farmers stated that they mainly restock when outbreaks are not occurring. The month of March was mentioned as restocking period.

**Figure 4:**
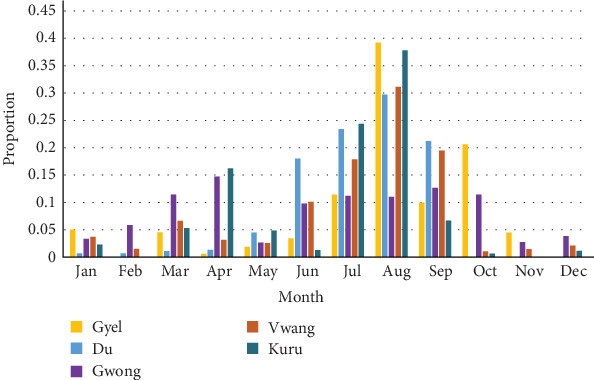
African swine fever occurrence as reported per district by farmer focus group discussions (FGDs) and other value chain FGDs in and around Jos, North Central Nigeria.

In comparing seasonality across the districts, farmer FGDs within Gwong districts reported increase in ASF outbreaks occurring mainly between March and April, which was a different pattern compared to the other districts (Figure 4). In Kuru, there was also a reported increase in ASF outbreaks in April, even though peak months were still July and August.

#### 3.4.1. Experience of ASF Outbreaks

Forty-two (78%) of the farmer FGDs reported at least one incidence of ASF outbreaks in the two previous years (2017–2019). In 12 farmer FGDs, all participants said they had not witnessed ASF in their farms/communities between 2017 and the time of the study. One FGD amongst those 12 reported having witnessed ASF 5 years before this study. In 29 (53.7%), 28 (51.9%), and 28 (51.9%) of the FGDs, farmers reported occurrence of outbreaks in 2017, 2018, and 2019, respectively. Out of the 29 FGDs that reported outbreaks in 2017, 20 FGDs reported mortality; while in 2018 and 2019, 21 FGDs, and 26 FGDs reported mortality, respectively. A total of 116 (25%), 81 (18%), and 102 (23%) individual farmers were affected amongst the participants for these years, respectively.

Both the farmer FGDs and trader FGDs discussed the consequences and impact of the outbreaks on their livelihoods, and both listed income loss. For the farmer FGDs, loss in income (otherwise used to rebuild pig pens), drop in pig prices, debts, and less manure from pigs were mentioned more than once. Participants also reported feeling discouraged subsequent to outbreaks. One farmer reported being hospitalized from shock after loss of pigs to ASF. The trader FGDs listed emergency sales by farmers resulting in lower price of pigs as a positive consequence of the outbreaks. Subsequent to such events, the participants reported scarcity of pigs to buy. The impact on the traders' livelihoods was mainly loss in income that would otherwise have been used for paying school fees and to purchase fertilizer. The participants reported that the occurrence of the disease sometimes affected the business relationship between the farmers and the butchers/middlemen negatively.

### 3.5. Practices

In 24 (56%) of the farmer FGDs, participants reported that they sold off sick pigs as soon as their pigs started to fall sick especially if they had heard of ongoing outbreaks in the neighborhood. Some farmers stated that they requested part of the pork to be given to them by butchers who came to buy dying pigs. Following the death of pigs due to suspected ASF, several disposal means were listed (Table 6). In 27 (50%) of the farmer FDGs, participants reported that they buried the carcasses. Further probing revealed burial sites to include around their houses, in the bush, or near the pig pens. It was a commonly reported practice to dump carcasses in bushes, caves, rivers, abandoned wells, and on hills/rocks. There were also reports of people that came and took away disposed carcasses to consume or sell. Some FGDs in Gyel district reported having a dedicated burial ground for pigs. In most FGDs, participants insisted that they did not eat pigs that die from disease, but in three (5%) farmer FGDs, participants reported consumption of dead pigs. However, in these three FGDs, farmers reported that dead pigs were cooked and given to other pigs or dogs. The majority of the trader FGDs (seven FGDS, 87%) reported that they had sold pigs suspected to be infected with ASF, while less than half of them had sold pork they knew was infected with ASF.

#### 3.5.1. Control Methods

Forty-five different control methods were listed by participants in the farmer FGDs. At least one control method was mentioned in each farmer FGD, with use of disinfectant being the most frequently mentioned. Control methods mentioned also included treating sick pigs with both conventional medication and local remedies like locust beans, baobab seed, and palm oil. Participants in 20 (37%) of the farmer FGDs said they were aware of other control methods besides the ones they had used in previous outbreaks. Use of disinfectant and “we advise the farmer” were the most frequently mentioned means of control of ASF (five FGDs, 62.5%) in the trader FGDs (Table S1). The actors at the Jos abattoir added that they do not visit farms that have suspected ASF outbreaks. This group of butchers said that they had previously attended an awareness program on ASF organized by the State Veterinary Department.

#### 3.5.2. Farming Practices

In 36 (67%) of the farmer FGDs, it was reported that replacement pigs were stocked from farms within the same district. 47 (87%) of farmer FGDs reported farm (pen) gate sales with some buyers coming from neighboring states, while 22 (45%) of the farmer FGDs reported home slaughtering, see current practices in Table 6.

## 4. Discussion

This study engaged farmers and other actors in the smallholder pig value chain in a participatory appraisal of ASF in Nigeria. Our study observed that pig farming was mostly motivated by profit-making. Most farmers provided housing, health, feed to livestock, and absolutely free roaming of pigs was rare. This is similar to observations by Rekwot et al. [29] and Adesehinwa et al. [1], and could be due to the peri-urban nature of the study locations [30]. The dominant management system could influence ASF occurrence with reduced pig-to-pig contact, production of healthier pigs, and increase in farmers' willingness to control disease in order to protect their investments.

Most participants had at least primary school education. This is similar to previous reports on demographics of pig farmers in Nigeria [1, 31], noting that this could facilitate further training within the area of ASF, as education has been reported to aid adoption of innovation [31]. Vice versa, lack of formal education has been reported to hinder adoption of farm biosecurity measures [32]. However, training, or increased knowledge alone are not linearly correlated to changes in biosecurity behavior or practice [33], with, for example, systemic factors having large impact on how smallholders can act [34].

Local names for ASF used by participants reflected the observed clinical signs. This is commonly seen in participatory studies [35]. Farmers used many different names, whereas less variation was observed with the traders, especially the butchers. This may be so because the traders were in a closer network, most belonging to an association, with some reporting previous participation in training on ASF. “Airborne” was indicated as the main route of spread by both farmers and traders, a few FGDs listed the technically established routes of spread, and some of the farmer FGDs were completely unaware of the mode of spread. This indicates specific training needs on ASF field transmission, prevention, and control. “Airborne” might seem like a plausible route of transmission if one does not know what a virus is, explaining this discrepancy between local and scientific knowledge in this regard [36]. This specific misconception can; however, be associated with a risk for continued disease spread. Previous studies show that believing that a disease is “airborne” is connected to feelings of powerlessness and helplessness concerning the disease threat, since if the disease is airborne there is nothing one can do to prevent spread [37]. As a consequence, less priority is given to investing and in implementing biosecurity for diseases that are (falsely) believed to be airborne [38]. Thus, it seems like specific knowledge-raising activities concerning how ASF is spread could be a way forward for improving biosecurity in the study setting.

The traders indicated that they were aware of their own role in disease spread, and most acknowledged their responsibility towards disease control, implicating themselves as drivers of disease transmission. The farmers' suspicion of traders' role in ASF spread corroborates the pivotal role of these actors which therefore must be intentionally engaged. It has previously been noted that preventing ASF outbreaks requires an active involvement of all stakeholders along the value chain [15, 39]. Levy et al. [40] and Ouma et al. [41] showed that pig butchers and traders were fundamental in this regard as they serve a large number of smallholders. Farmer groups have been suggested as a way to share information and knowledge, build capacities, as well as create common ownership of disease problems and solutions [42–45].

Both categories of FGD groups experienced negative impact of ASF. According to the results, the traders (especially the middlemen and butchers) benefited at the start of the outbreak by buying pigs at a cheaper rate, but later experienced scarcity of pigs, affecting their long-term income. This agrees with Fasina et al. [46] who reported traders taking initial advantage of oversupply during outbreaks, but in succeeding months suffer from high prices due to scarcity of pigs. Kagira et al. [47] also reported irregular supply of pigs after ASF outbreaks additionally had an impact on food security. In Uganda, Muhangi et al. [48] reported traders buying pigs from infected farms at reduced prices, causing risk for disease spread.

The consensus by all participants regarding the absence of wild pigs confirms the sole occurrence of the domestic pig epidemiological cycle in this region, as previously reported by several authors [17, 25]. Furthermore, in this study, ASF occurrence did not seem to be associated with peaks in pig or pork sales. In this study, seasonal peaks in occurrence of ASF instead coincided with the rainy season (July–August), which is also the cropping season, during which pigs were confined at all times. This is different from a similar study in Uganda, where temporal overlap was seen between peaks in sales and ASF outbreaks [26]. In Tanzania; however, Fasina et al. [46] showed similar overlap of peaks with rainy season and ASF as in our study. This indicates the need to understand the epidemiology of ASF in context to the system, paying close attention to local disease drivers and risks for disease spread. Studies on historic outbreaks in Nigeria reported ASF to be present throughout the year [17, 49]. Another study in the southern part of Nigeria indicates dry season as a risk factor for ASF and suggests scarcity of feed and movement of pigs as drivers of disease transmission [19]. Increased ASF occurrence during the crop cultivating season could possibly be linked to the use of pig manure in the fields, with manure acting as a potential source of virus if coming from infected pigs. ASF virus survival times at the temperatures experienced during the crop cultivation season in Plateau State (18–22°C) are; however, short (2–7 days) [50]. Shallow burial as well as dumping of carcasses (during ASF outbreaks) in bushes, rivers, abandoned wells, and on hills were commonly reported. This is similar to reports in Cameroon [51], where carcasses could be seen dumped along roads and stream banks. In Nigeria, Owolodun et al. [17] confirmed that carcasses found dumped in trenches during outbreaks were ASF virus positive. Rain/water may also play a role in spreading ASF, washing away blood and offal of slaughtered, infected pigs. Unsafe handling of blood and offal, and slaughtering of sick pigs was evident from our farm observations. Carcass disposal is an important practice that should be an intervention point to reduce ASF spread, but for which traditions, cultural taboos, food security issues, land, labor, costs, and infrastructure can hinder change [34, 52]. One example of a safe carcass handling practice that could be replicated was a dedicated burial site reported in one of the districts. In addition, home slaughter as seen in this study might spread disease if sick, possibly ASF-infected pigs are slaughtered, and waste is disposed in a way that naïve pigs can get access. Furthermore, farm gate sales and close proximity to slaughter slabs and abattoir were shown by Fasina et al. [10] to be high-risk factors for presence and spread of ASF. The practice of selling sick pigs to butchers was frequently reported as a coping mechanism upon suspected outbreaks. This further supports both direct and indirect (farm–farm) transmission.

The study had some limitations and possible biases. No strict case definition of ASF was used in this study; instead, disease descriptions were assigned to being ASF/not ASF based on a syndromic disease description. Despite that, we remain confident that most (if not all) of the diseases discussed by the participants in this study were ASF. In this regard, it is well established that smallholder farmers and pastoralists are knowledgeable about animal diseases that are important for them and that their disease descriptions can be used for participatory disease surveillance [22, 53, 54]. This was, for example, used in the global campaign to eliminate Rinderpest [55]. The absence of classical swine fever (CSF) in Nigeria as a differential diagnosis [56] aids in confirming cases of disease with high case mortality as ASF. ASF has further been confirmed in Jos both in the past [17, 49] and recently [21]. The purposive selection of villages and the nonrandomized sampling of households mean that care should be taken when drawing generalized inferences or extrapolating the study results to other populations. All groups were not facilitated by the same person; however, the team was trained together before the study commenced, and not more than four different facilitators were used throughout the study in order to curb interview bias. Power dynamics can hinder equal participation [53]. In this study, the facilitators were trained to balance consensus versus heterogeneity, and efforts were made to record all opinions, even though consensus was sought. FGDs were mostly held in Hausa, but the analysis was made from transcripts translated into English. This could have led to the loss of information.

In summary, this study contributes valuable insights to the current discourse on ASF and its control in the region, emphasizing the importance of context-specific approaches and to engage various stakeholders in a participatory approach. Increased awareness of how different stakeholders recognize the disease, routes of disease spread and control measures is important for designing a prevention and control plan that can be implemented in the field. The perceived temporal occurrence of the disease needs further investigation. Participatory planning, including all value chain actors and local adoption of biosecurity measures [57], could be a logical next step for control of ASF in the study area [52].

## 5. Conclusions

According to the results, indirect transmission is driving ASF spread in this local setting. This requires further investigation to find out the exact modes of transmission and following, how this can be prevented. It is also critical to address the knowledge gap concerning modes of spread, especially the misconception of airborne spread, in order to increase the priority to be given to biosecurity. Furthermore, the role of traders/butchers in the spread was emphasized, indicating the need to involve all value chain actors to tailor control interventions that are feasible and suitable to the context and for all concerned. Results of this study can be used to guide the development of interventions for the control of ASF in the smallholder pig value chains in Nigeria.

## Figures and Tables

**Table 1:** Focus group discussions (FGDs) conducted in a study on knowledge, perceptions, and practices concerning African swine fever (ASF) in smallholder pig production in North Central Nigeria in 2019.

District	Interview site	Connected slaughter/pork point^a^	Farmer FGDs (number)	Trader FGDs (number)
Gwong	Dong, Kabong, Tudun Wada	Jos abattoir	15	3^b^
Du	Du, Rabin Du, Kufang	Jos abattoir, Ladura	9	1
Vwang	Vwang, Turu Lah, K/Vom	Vwang	13	2
Kuru	Kuru	Kuru	6	2
Gyel	Rasot, Gura Riyom, Rantya	Jos abattoir, Kuru	11	0^c^
Total	—	—	54	8

^a^Connected points are based on the activities in the value chain and association to selected interview sites.

^b^Three FGDs were organized in the Jos abattoir. The same traders operate in Jos abattoir and in Gwong, Gyel, and Du districts.

^c^The same traders operate in Gyel, Jos abattoir, and in Kuru.

**Table 2:** Questions and participatory epidemiology (PE) tools used in focus group discussions (FGDs) on knowledge perceptions and practices concerning African swine fever (ASF) in smallholder pig production conducted in North Central Nigeria in 2019.

Question	Tool	Farmer FGDs	Trader FGDs
Background information			
Production system	Hand count	X	—
Housing and management system	Hand count	X	—
Activities in the pig value chain	Hand count	—	X
Number of pigs owned or slaughtered/traded per day	Hand count	X	X
Knowledge			
Indigenous name of ASF	Listing	X	X
Clinical signs of ASF	Listing	X	X
Sure to recognize ASF	Hand count	X	X
Age/breed classes affected	Hand count	X	—
Have seen ASF signs in pigs/products	Hand count	—	X
Routes for ASF spread	Listing	X	X
Business-related routes for ASF spread	Listing	—	X
Seasonality of ASF outbreaks and related factors	Seasonal calendar/proportional piling	X	X
Perceptions			
Perceived public health risks with ASF	Hand count	X	X
Perceived public health risks with other pig diseases	Hand count	X	X
Know a model farmer	Hand count	X	X
Reasons for success of model farmer	Listing	X	X
Perceived responsibility to disease control in relation to value chain activities	Listing	X	X
Socioeconomic impacts of ASF outbreak	Listing	X	X
Outbreaks			
Number of outbreaks/affected farmers	Hand count	X	—
Practices relating to outbreaks			
Sale/trade in pigs or products with ASF signs	Hand count	X	X
Disposal of sick pigs/carcasses	Hand count and listing	X	—
Back in business after ASF outbreak	Hand count	X	X
Control methods used	Listing	X	X
Aware of other control methods than those used	Hand count	X	X
Other control methods than those used	Listing	X	X
Obstacles to prevent using control methods	Listing	X	X
Incentives for using control methods	Listing	X	X
Current practices post outbreaks			
Source of pigs for stocking and restocking	Hand count and listing	X	—
Sales point	Hand count and listing	X	—
Slaughter point	Hand count and listing	X	—
Transport means for movement of pigs	Hand count and listing	X	—

**Table 3:** Demographic information of participants in focus group discussions (FGDs) on knowledge perceptions and practices concerning African swine fever (ASF) in smallholder pig production conducted in North Central Nigeria in 2019.

Question	Farmer FGDs (number, [%])^a^	Trader FGDs (number, [%])	Both categories of FGDs (number, [%])
FDGs			
All male	5 (9)	2 (25)	7 (11)
All female	3 (6)	1 (2)	4 (6)
Mixed	46 (85)	5 (62)	51(82)
Total	54	8	62
Participants			
Male	242 (53)	35 (55)	277 (54)
Female	210 (47)	29 (45)	239 (46)
Total	452	64	516
Age (in years); mean (range)	44 (19–100)	38 (18–85)	43 (18–100)
Highest educational level			
None	68 (15)	6 (9)	74 (14)
Primary school	133 (29)	24 (38)	157 (30)
Secondary school	119 (26)	32 (50)	151 (29)
Tertiary institution	111 (27)	2 (3)	113 (22)
Production system			
Breeder	24 (5)	N/A	—
Grower	69 (15)	N/A	—
Both breeder and grower	359 (79)	N/A	—
Type of breed			
Local	327 (72)	N/A	—
Cross/improved	81 (18)	N/A	—
Exotic/foreign	72 (1)	N/A	—
Housing			
Free range	2 (0)	N/A	—
Tethered	23 (5)	N/A	—
Housed	426 (95)	N/A	—
Management system^b^			
Intensive	332 (74)	N/A	—
Semi-intensive	119 (26)	N/A	—
Extensive	0	N/A	—
Activities within the pig value chain^c^			
Middleman (buys and sells live pigs)	N/A	30 (47)	—
Butcher/trader	N/A	37 (58)	—
Owner of pork kiosk	N/A	16 (25)	—
Owner of pork joint	N/A	6 (9)	—
Owner/breeder of pigs	N/A	20 (31)	—
Others (e.g., slaughter only)	N/A	23 (36)	—

^a^Number, (%) is valid for all rows apart from age.

^b^Management system refers to the farmer's provision of feed, health care, and housing to pigs.

^c^Some of the other value chain actors were involved in more than one activity, hence over 100% summation.

**Table 4:** “Knowledge” from focus group discussions (FGDs) conducted on knowledge perception and practices toward African swine fever in the smallholder pig production in North Central Nigeria in 2019.

Question	Farmer FGDs (number, [%]) *n* = 54		Trader FGDs (number, [%]) *n* = 8	
Names for ASF	Robi alede (berom)	5 (9)	Siriri^a^	3 (37)
	Swine fever	3 (6)	Swan^a^	3 (37)
	Ciwon alede (hausa)	4 (7)	Baddo^a^	2 (25)
	Skin disease	4 (7)	—	—
				
Clinical signs of ASF	Inappetence	39 (72)	Inappetence	8 (100)
	Red skin	38 (70)	Red spots on skin	8 (100)
	Death within 2–7days	31 (57)	Weakness	4 (50)
	Weakness	24 (44)	Shivering	4 (50)
	Shivering	21 (39)	—	—
				
Seen ASF signs in pigs/encountered ASF before				
Yes	43 (80)	—	8 (100)	—
No	1 (2)	—	—	—
No response	10 (18)	—	—	—
Seen ASF signs in pork	N/A	—	—	—
Yes	—	—	8 (100)	—
No	—	—	—	—
Sure to recognize ASF				
Yes	24 (44)	—	8 (100)	—
No	8 (14)	—	0	—
No response	22 (42)	—	—	—
Age affected				
Piglet	2 (4)	—	N/A	—
Adult	6 (11)	—	N/A	—
Both	34 (63)	—	N/A	—
Breed affected				
Local	4 (7)	—	N/A	—
Cross/improved	1 (2)	—	N/A	—
Both	32 (60)	—	N/A	—
				
Routes for ASF spread	Airborne	20 (37)	Airborne	7 (88)
	Visiting infected farms	10 (19)	Farm to farm	5 (63)
	Pig to pig contact	10 (19)	Pig to pig contact	5 (62)
	Free range pigs	8 (15)	Flies	2 (25)
	Butchers' movement	7 (13)	—	—
	Improper disposal of dead pigs	7 (13)	—	—
	Ticks	6 (11)	—	—

*Note*: Siriri = shivering; Baddo = bad/mean disease; swan = local pronunciation of swine.

^a^Names used by butchers to identify ASF based on their perception of ASF.

**Table 5:** Perceptions from focus group discussions (FGDs) conducted on knowledge perception and practices toward African swine fever in the smallholder pig production value chain in North Central Nigeria in 2019.

Question	Farmer FGDs (number, [%]) *n* = 54	Trader FGDs (number, [%]) *n* = 8
Perceived public health risks for ASF		
Yes	13 (24)	—
No	11 (22)	5 (62)
Not aware	13 (24)	3 (37)
No response	17 (31)	—
Perceived public health risks for other pig diseases		
Yes	12 (22)	5 (62)
No	3 (5)	—
Unable to answer/not aware	39 (72)	3 (37)
Know a model farmer		
Yes	14 (26)	5 (62)
No	40 (74)	3 (37)
Reasons given for success of model farmer		
Hygiene/clean environment	2 (4)	4(50)
Big farm with restriction to visitors	2 (4)	—
Water is available	2 (4)	—
Good breeds	2 (4)	—
Good feeding	—	2(25)
Aware that business activity can spread disease		
Yes	N/A	5 (62)
Not aware/known	N/A	3 (37)
Responsibility for controlling ASF in relation to pig value chain activity		
Yes	N/A	6 (75)
No	N/A	2 (25)

**Table 6:** Practices from focus group discussions (FGDs) conducted on knowledge perception and practices toward African swine fever in smallholder pig production in North Central Nigeria in 2019.

Question	Farmer FGDs		Trader FGDs *n* = 8 (number [%])
	*N* ^a^	(number [%])	
Have sold pigs with signs of ASF	43	—	—
Yes	—	24 (56)	7 (88)
No	—	19 (44)	1 (13)
Have sold pork with signs of ASF	—	N/A	—
Yes	—	—	3 (38)
No	—	—	2 (25)
No response	—	—	3 (38)
Back in business after ASF	54	—	—
Yes	—	52 (96)	8 (100)
No	—	2 (4)	—
Control methods used	54	—	—
Disinfection	—	17 (31)	5(63)
Restriction of access	—	11 (20)	—
Treatment with local remedies^b^	—	13 (24)	—
Confinement	—	10 (18)	—
Call a vet or animal health worker	—	11 (20)	—
Use of work clothes	—	—	3 (37)
Wash tables used for pork	—	—	5 (62)
Aware of other control methods besides those used	54	—	—
Yes	—	20 (37)	4 (50)
No	—	14 (25)	4 (50)
No response	—	20 (37)	—
Disposal of dead pigs during outbreaks	54	—	—
Dumped in river/stream	—	9 (16)	N/A
Consumption	—	3 (5)	N/A
Dumped in cave/rocks/hills	—	10 (18)	N/A
Dumped in bushes	—	11 (20)	N/A
Dumped in abandoned well	—	8 (15)	N/A
Buried	—	27 (50)	N/A
Dumped in bin	—	2 (4)	N/A
Dumped in mining pond	—	2 (4)	N/A
Burning	—	2 (4)	N/A
Current practices (farmers)	—	—	N/A
Source of pigs for stocking	49	—	—
From other farms within the district	—	36 (67)	N/A
Market	—	2 (4)	N/A
Others (within and outside district)	—	10 (18)	N/A
Pig sales point	49	—	—
Farm gate	—	47 (87)	N/A
Market	—	2 (4)	N/A
Others	—	1 (2)	N/A
Pig slaughter point	48	—	—
At home	—	22 (41)	N/A
At slab/abattoir	—	11 (21)	N/A
Others (emergency slaughter only)	—	15 (28)	N/A
Transport means for movement of pig	45	—	—
Motor bike	—	7 (13)	N/A
Car	—	6 (11)	N/A
Both	—	32 (59)	N/A

^a^Number of FGDs answering the particular question.

^b^Local remedies include locust beans, baobab seed, and palm oil.

## Data Availability

The data that support the findings of this study are available from the corresponding author upon reasonable request.

## Supporting Information

Supporting Information Table S1: Extensive list of answers for clinical signs, routes of ASF spread, and control methods given by farmers and other value chain actors in the FGDs.
