# Supplementary material for: Knowledge, Perception, and Practices Concerning African Swine Fever in Smallholder Pig Value Chain in North Central Nigeria: Implications for Adaptation of Prevention and Control
Source: Transbound Emerg Dis. 2025 Aug 28;2025:5582374. doi: 10.1155/tbed/5582374 (PMC12411033; doi:10.1155/tbed/5582374)
Supplement: Supporting Information — Table S1: Extensive list of answers for clinical signs, routes of ASF spread, and control methods given by farmers and other value chain actors in the FGDs. [file 5582374.f1.docx]

**Table S1. Extensive list of answers for clinical signs, routes of African swine fever spread and control methods given by farmers and other value chain actors in the FGDs.**

| **Question** | **Farmer FGDs** | **Trader FGDs** |
| --- | --- | --- |
| **Local names given for ASF** | Robi alede, Swine fever, Ciwon alede, Skin disease, Swan, Roh alede, Baddo, Ro-gul, Rochin (meaning flies)  **Names listed by only one FGD:**  Ntang, Swan fever, Malaria fever, Zazabi, 2^nd^ HIV, Aloba | \| Siriri, Swan, \| \| --- \| \| **Names listed by only one FGD:**  Baddo \| |
| **Clinical signs of ASF** | Off feed, red Skin, shivering, weakness, Death in 2-7days, fleas, lie down, raised hair, high temperature, salivation, diarrhoea, dullness/depression, rough hair, itching of body, bleeding from sore, emaciation, watery/teary of eyes, red eyes, standing and falling, paralysis (unable to stand), rashes, laboured breathing, eyes and mouth discharge, scaly skin/rough skin, vomiting  **Signs listed by only one FGD:**  Bleeding from nose, restless, struggling to breathe, hair loss, seed like substance at slaughter, black spots, discharge from mouth/nose, stunted growth, abortion, does not breast feed, change in colour, bleeding from anus, bleeding from sweat pores, they isolate themselves | No appetite, weakness , black spots, shivering, red spot/skin, fever, sudden death  **Signs in meat**  Blood in lap, haemorrhage in organs, black spot in liver, tasteless meat  **Signs listed by only one FGD:**  Lumpy faeces, abortion, weight loss, red ear, frothy mouth, bleeding from the nose;  No fat in carcass, perforation of liver |
| **Routes for ASF spread** | Unknown, airborne, mating, dirty pens, contamination, flies, vet movement, butchers movement, visiting infected farms, ticks/lice, birds, swill feeding, free range, collecting food left over, improper disposal, joining pigs with infected pigs, slaughter, pigs, feeding trough, during delivery, middlemen, contact with farmers, contact with infected pig, consuming of infected pork, contact with dead pig, pig to pig contact, eating infected pork, pig eating human faeces, man to pig, farm to farm, poor hygiene  **Routes listed by only one FGD:**  cold weather, change in weather, wind, from abattoir, cutting grass, people buying pigs, dirty water, fomites, transport of slaughtered pig, workers cloth, feed infected offals, water and rivers with dead pig | Air, farm to farm, pig to pig, flies  **Routes listed by only one FGD:**  Heat, slaughter point, change of weather, eating infected meat, poor hygiene, infected clothes |
| **Business related routes** | Not applicable | Farm to farm, unclean clothing, selling infected pigs, bad handling of slaughtered pigs, consuming infected pork, wrong disposal, mixing of healthy and infected pigs, bad handling of blood and offals, reckless entry into farms |
| **Control methods used** | Wash pen with disinfectants, restriction of access to pen, call a vet/health worker, treatment with local remedy, confinement of pigs/intensive management, stop visiting farms, burying infected animal, isolation/quarantine, stop eating pork, wash pen regularly, weed the environment, labour for vet and staff, need farmers to form association, dip /disinfection, use of Indian hemp as medication, sell pigs off, improve hygiene, close pen with net, better feed, wash pen with detergent, daily washing of pen  **Control methods listed by only one FGD:**  Treatment with antibiotics, depopulate, fence pen (pig house), does not eat pork indiscriminately, roof house with net, vet to change cloth, farmer changes cloth after farm activity, avoid getting grass, provide clean water, avoid selling dead pigs, buying commercial feed, wash body of pigs, does not eat sick pigs, tincture of iodine in water, prevent pig to pig contact, wash 3 times a week, avoid butcher, wash feed trough, pen was left to fallow, close observation, avoid mating, bury dead pigs | Advice farmers, isolation of infected pig, use of izal/alum, uniform, wash feeder/table, restriction  **Control methods listed by only one FGD:**  Have a trade association, spray “white maggi” (food seasoning), spray kerosene and ash, slaughter infected pig, burial/disposal |

Most of the names describe clinical signs. Translation to English when appropriate: Ciwon alede/Robi alede/ Roh alede = disease of pig; Rochin = flies, Zazabi = fever, Aloba = plague
